# Improved FGF21 Sensitivity and Restored FGF21 Signaling Pathway in High-Fat Diet/Streptozotocin-Induced Diabetic Rats After Duodenal-Jejunal Bypass and Sleeve Gastrectomy

**DOI:** 10.3389/fendo.2019.00566

**Published:** 2019-08-30

**Authors:** Qiaoran Liu, Shuo Wang, Meng Wei, Xin Huang, Yugang Cheng, Yi Shao, Pingtian Xia, Mingwei Zhong, Shaozhuang Liu, Guangyong Zhang, Sanyuan Hu

**Affiliations:** ^1^Department of General Surgery, Shandong Provincial Qianfoshan Hospital, Shandong University, Jinan, China; ^2^Department of General Surgery, Qilu Hospital of Shandong University, Jinan, China

**Keywords:** duodenal-jejunal bypass, sleeve gastrectomy, FGF21, type 2 diabetes mellitus, lipid metabolism

## Abstract

**Objective:** Bariatric surgery can profoundly improve glucose and lipid metabolism in diabetic rats. Fibroblast growth factor 21 (FGF21) is an important hormone with multiple metabolic beneficial effects. Alteration in serum FGF21 level after bariatric surgery has been reported with conflicting results. Here, we investigated the effect of bariatric surgeries on FGF21 expression and sensitivity.

**Methods:** We performed duodenal-jejunal bypass (DJB), sleeve gastrectomy (SG) and sham surgery in diabetic rats induced by high fat diet and streptozotocin. Metabolic parameters, including body weight, food intake, glucose tolerance, and lipid profiles, were monitored. FGF21 levels in both serum and liver were measured after surgery. FGF21 signaling pathway including FGF receptor 1 (FGFR1), β-klotho (KLB), and phosphorylated extracellular signal-regulated kinase 1/2 (ERK1/2) was detected in the liver and white adipose tissue (WAT). We also determined FGF21 sensitivity post-operatively by acute recombinant human FGF21 injection. Oral glucose tolerance test (OGTT) and insulin tolerance test (ITT) were conducted immediately after FGF21 injection. Serum triglyceride (TG) and non-esterified fatty acid (NEFA) were measured and the mRNA levels of early growth response 1 (Egr1) and c-Fos in the liver and WAT were detected after FGF21 injection.

**Results:** Improvements in glucose tolerance, insulin sensitivity, and lipid profiles were observed after bariatric surgeries along with ameliorated lipid metabolism in the liver and WAT. Serum and hepatic FGF21 levels decreased in both DJB and SG groups. FGFR1 and phosphorylated ERK1/2 levels increased in both DJB and SG groups 8 weeks after surgery. The expression of KLB was downregulated only in the WAT after DJB and SG. Significant alteration of OGTT and ITT were observed after acute FGF21 administration in DJB and SG groups. Serum TG and NEFA in DJB and SG groups also decreased after FGF21 administration. And increased mRNA levels of Egr1 and c-Fos were detected in the liver and WAT after DJB and SG surgeries.

**Conclusions:** DJB and SG surgeries can downregulate hepatic expression of FGF21, restore FGF21 signaling pathway and improve FGF21 sensitivity in high-fat diet/streptozotocin-induced diabetic rats.

## Introduction

Type 2 diabetes mellitus (T2DM) is one of the most prevalent metabolic diseases in the world and a threat to human health (1). Traditional treatment strategies for controlling T2DM and associated complications, including calorie restriction, medication, and lifestyle modification, have failed to yield satisfactory results (2). Bariatric surgery has been shown to be highly effective for T2DM remission (3, 4), of which Roux-en-Y gastric bypass (RYGB) and sleeve gastrectomy (SG) are now the most commonly performed procedures (5). Duodenal-jejunal bypass (DJB) was originally designed to investigate the mechanisms of intestinal reconstruction of RYGB (6) which has comparable metabolic benefits without causing significant weight loss (7). However, the complex mechanisms behind the metabolic changes elicited by bariatric surgery have not been thoroughly explored.

Fibroblast growth factor 21 (FGF21) is a novel member of the FGF superfamily which is considered to be an important regulator in glucose and lipid metabolism (8, 9). It's predominantly produced in the liver with lower expression in pancreas, adipose tissue and skeletal muscle (10). Studies with rodents and humans have confirmed that the administration of FGF21 or its analog in obese and diabetic state lowers body weight and improves insulin sensitivity (11, 12). Further experiments revealed that FGF21 could promote fatty acid (FFA) oxidation and suppress lipogenesis in the liver (13, 14) and inhibit lipolysis in the white adipose tissue (WAT) (15, 16). FGF21 activates FGF receptor (FGFR) in association with an essential co-receptor called β-klotho (KLB) and subsequently phosphorylates the extracellular signal-regulated kinases 1 and 2 (ERK1/2) (17, 18).

Although FGF21 performs several beneficial functions, its serum level is known to be increased in obesity and diabetes (19, 20), suggesting a potential FGF21-resistant state (21). In support of this hypothesis, the expression of KLB and FGFR was downregulated in the liver and WAT in both obese and diabetic state. And the phosphorylation of ERK1/2 and the activation of downstream genes such as Egr1 and c-Fos were also impaired in obese and diabetic state (21–24).

The effect of bariatric surgery on serum FGF21 level has been studied in several research groups with contradictory results. The level of FGF21 after RYGB was reported to increase (25, 26), decrease (27, 28), or remain unchanged (29, 30). However, downregulated expression of circulating FGF21 was confirmed after SG surgery (31). To our knowledge, the influence of DJB on the serum level of FGF21 has not been reported. And the effect of either DJB or SG on FGF21 signaling pathway and FGF21 sensitivity is currently unknown.

In the present study, DJB, SG, and sham surgical procedures were performed in a diabetic rat model induced by high-fat diet (HFD) and low-dose streptozotocin (STZ). We aimed to investigate the serum levels and hepatic expression of FGF21 after DJB and SG surgeries, and the effect of DJB and SG on FGF21 signaling and sensitivity.

## Materials and Methods

### Animals

Wistar rats (8-week-old, 200 g on average) used for this study were purchased from the Laboratory Animal Center of Shandong University (Jinan, China) and housed in independent ventilated cages. The animals had free access to food and tap water. After 1 week of acclimatization, rats were fed with chow diet (15% fat, Laboratory Animal Center of Shandong University, China) or HFD (40% fat, Huafukang Biotech, China) for 4 weeks. Rats fed with HFD were administered with a single intraperitoneal injection of low-dose STZ (35 mg/kg; Sigma, USA) to induce insulin resistance and hyperglycemia, as previous described (32), and randomly divided into sham group, DJB group and SG group. All animal protocols were approved by the Ethics Committee on Experimental Animals of Qilu Hospital of Shandong University and performed in accordance with the National Institutes of Health Guidelines.

### Surgical Procedures

Diabetic rats were fed with a low-residue diet (10% Ensure, Abbott, USA) for 2 days and fasted for 12 h before surgery. Anesthesia was achieved with 2% isoflurane. Rats were allowed to access water 2 h after operation and 10% Ensure (Abbott, USA) 24 h after surgery for 3 days, followed by a standard chow diet.

#### Duodenal-Jejunal Bypass

DJB was performed as previously described (33). The duodenum was transected at 1 cm distal to the pylorus, and the stump was closed with a 7-0 silk suture (Ningbo Medical Needle, China). After jejunum transection at 10 cm distal to the Treitz ligament, duodenojejunal anastomosis was performed to connect the proximal end of the duodenum to the distal end of the jejunum. The proximal end of the jejunum (biliopancreatic limb) was anastomosed to the alimentary limb, 15 cm distal to the duodenojejunal anastomosis.

#### Sleeve Gastrectomy

As previously reported (34), the omentum was dissected to reveal the greater curvature of the stomach. Related blood vessels including short gastric vessels, related gastroepiploic vessels, and the branches of left gastric vessels in the greater curvature were ligated and transected. The gastric fundus and a large portion of the gastric body (70% of total stomach) were resected. The residual stomach was stitched with a 5-0 silk suture (Ningbo Medical Needle, China).

#### Sham Surgery

The sham surgery was a laparotomy to expose the intestines, stomach and esophagus. And the abdominal wall was closed afterwards. The operative time was prolonged to mimic the degree of surgical and anesthetic stress in DJB and SG groups.

### Oral Glucose Tolerance Test (OGTT) and Insulin Tolerance Test (ITT)

After fasting the rats for 12 h, the blood glucose was collected at baseline and 15, 30, 60, 90, and 120 min after the administration of 20% glucose (1 g/kg) by an intragastric gavage for OGTT or after administration of human insulin (0.5 IU/kg) by an intraperitoneal injection for ITT. ITT was performed 2 days after OGTT to ensure full recovery. Glucose concentration was determined with glucometer (Roche Diagnostics, Germany). Serum glucagon-like peptide-1 (GLP-1) concentration was measured with multi-species GLP-1 total ELISA kit (Millipore, USA).

### Blood Sampling and Analysis

After fasting the rats for 12 h, blood samples were collected from retrobulbar venous plexus with capillary tubes under light ether anesthesia post-operation at 2 and 8 weeks. Blood serum was isolated by centrifugation and stored at −80°C for analysis. Serum triglyceride (TG), cholesterol (CHO), non-esterified fatty acid (NEFA), high-density lipoprotein (HDL), and low-density lipoprotein (LDL) levels were measured with the Roche Cobas 8000 system. Serum creatinine and blood urine nitrogen (BUN) were measured using an automatic biochemistry analyzer at the laboratory of Qilu Hospital of Shandong University. Serum insulin level was measured using Rat/Mouse Insulin ELISA Kit (Millipore, USA). Serum FGF21 concentration was measured with Mouse/Rat FGF-21 Quantikine ELISA Kit (R&D Systems, USA). The homeostasis model assessment of basal insulin resistance (HOMA-IR) was calculated as fasting blood glucose (FBG, mmol/L) × fasting insulin (mIU/L)/22.5 (35).

### Histology and Staining

Liver and epididymis adipose tissues were harvested and fixed in 4% paraformaldehyde for 48 h. Fixed tissues were embedded in paraffin, sectioned for the subsequent hematoxylin-eosin (HE) staining. Adipocyte sizes were calculated with ImageJ software (National Institutes of Health, USA). Immunohistochemistry (IHC) of FGF21 (1:250, Abcam, USA) in liver sections was also performed.

### Liver TG Content Analysis

Liver samples (100 g) were homogenized and centrifuged at 10,000 × g for 10 min at 4°C. The TG content was colorimetrically analyzed with Triglyceride Assay Kit (Solarbio, China) as per the manufacturer's instruction.

### Real-Time Quantitative Polymerase Chain Reaction (RT-qPCR)

Total RNA was extracted from the liver, epididymis adipose tissue and soleus with TRIzol Reagent (Thermo, USA) and treated with DNase (Invitrogen, USA). The total RNA was reverse transcribed into cDNA using a reverse transcription kit (Promega, USA) according to the manufacturer's protocol. The cDNA was subjected to real-time PCR quantitation with SYBR Green Real-Time PCR Master Mix (Bio-Rad, USA) on a Bio-Rad Real-Time PCR system (Bio-Rad, USA). β-Actin was used as an internal control. The cycle threshold (Ct) of amplification was determined, and the expression scores were quantified with the 2^−ΔΔ*Ct*^ method. The primers used in this study are shown in Table 1.

**Table 1:** Primers for RT-qPCR.

**Gene**	**Forward primer (5^′^-3^′^)**	**Reverse primer (5^′^-3^′^)**
Acc	CTTCGCCAGCAGAATTTGTTAC	TGCGGAACATTTCATAAGACCA
Atgl	TTCAAGTTTCCTTGCAGAGT	CTCCCAAACTGACCCTTAAA
c-Fos	GGGACAGCCTTTCCTACTACCA	GGAGATAGCTGCTCTACTTTGCC
Cpt1α	AGAGGATGGACACTGTAAAGGAGA	CCGAAAGAGTCAAATGGGAAGG
Egr1	GAGCGAACAACCCTACGAGCA	TGAGGATGAAGAGGTTGGAGGG
Fasn	TGATGATTCAGGGAACGGGTAT	GACCGAGTAATGCCGTTCAGTT
Fgf21	GGGTCAAGTCCGACAGAGGTAT	ATCAAAGTGAGGCGATCCATAGA
Hsl	TCACGCTACATAAAGGCTGCT	CCACCCGTAAAGAGGGAACT
Scd1	ATCGCCCCTACGACAAGAAC	AGGAACTCAGAAGCCCAGAAC
Srebp-1c	ACCCTGTAGGTCACCGTTTCTTC	TGGTAGCCATGCTGGAACTGAC
β-actin	TGCTATGTTGCCCTAGACTTCG	GTTGGCATAGAGGTCTTTACGG

*RT-qPCR, real-time quantitative polymerase chain reaction; Acc, acetyl-CoA carboxylase; Atgl, adipose triglyceride lipase; Cpt1α, Carnitine palmitoyltransferase I; Egr1, early growth response gene 1; Fasn, fatty acid synthase; Fgf21, fibroblast growth factor 21; Hsl, hormone-sensitive lipase; Scd1, stearoyl-CoA desaturase 1; Srebp-1c, sterol-regulatory element binding protein 1c*.

### Western Blot Analysis

Total proteins from the liver and epididymis adipose tissue were extracted with a protein extraction kit (BestBio, China) and quantified with a bicinchoninic acid (BCA) protein assay kit (Beyotime, China). Equal quantities of proteins from each group were loaded on 8–12% sodium dodecyl sulfate polyacrylamide gel (SDS-PAGE) and separated by electrophoresis. Proteins were transferred onto polyvinylidene fluoride (PVDF) membranes (Millipore, USA) and the membranes were blocked with 5% non-fat milk for 2 h. The membranes were incubated with primary antibodies overnight at 4°C, followed by treatment with horseradish peroxidase (HRP)-conjugated secondary antibodies for 1 h. Primary antibodies used for western blotting are as follows: FGF21 (1:1,000, Abcam, USA), KLB (1:1,000, Sigma, USA), FGFR1 (1:1,000, Abcam, USA), ERK1/2 (1:1,000, Cell Signaling Technology, USA), p-ERK1/2 (1:1,000, Cell Signaling Technology, USA), and glyceraldehyde 3-phosphate dehydrogenase (GAPDH; 1:1,000, Proteintech, China). The protein bands were visualized using an enhanced chemiluminescence (ECL) solution (Millipore, USA), and the reactive signals were quantified with ImageJ software (National Institutes of Health, USA).

### Acute Administration of Recombinant Human FGF21

After 12 h fasting, rats were administrated with vehicle (phosphate-buffered saline, PBS) or recombinant human FGF21 (rhFGF21, 100 μg/kg, R&D System, USA) 8 weeks after surgery by a single intraperitoneal injection. OGTT and ITT were performed immediately after FGF21 administration. After rest for 1 week, the same procedure of administration was performed again. And blood samples, liver and epididymis adipose tissue were harvested 2 h later for further analysis.

### Statistical Analysis

Quantitative data are presented as mean ± standard deviation and analyzed using SPSS software (ver. 22.0; SPSS Inc., USA). Area under the curves for OGTT (AUC_OGTT_) and ITT (AUC_ITT_) were calculated by trapezoidal integration. Intergroup comparisons were carried out with student's *t*-test or one-way analysis of variance (ANOVA) followed by Bonferroni or Dunnett's T3 correction. *P* < 0.05 was considered statistically significant in all cases.

## Results

### Body Weight and Food Intake After Surgery

**Figure 1:**
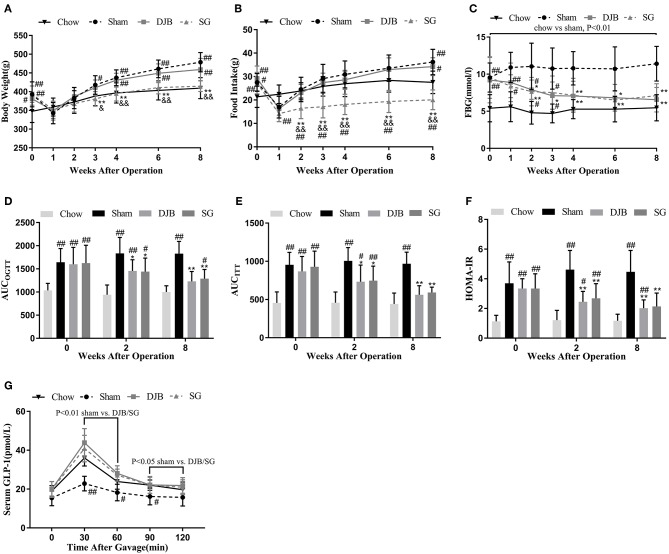
Effect of DJB and SG on body weight, Food intake, and glucose homeostasis in diabetic rats. **(A)** Body weight, **(B)** Food intake, and **(C)** FBG before and after surgery. **(D)** AUC_OGTT_, **(E)** AUC_ITT_, and **(F)** HOMA-IR before operation and 2 and 8 weeks post-operation. **(G)** Serum GLP-1 measured 8 weeks after surgery. DJB, duodenal-jejunal bypass; SG, sleeve gastrectomy; FBG, fasting blood glucose; AUC, area under the curve; OGTT, oral glucose tolerance test; ITT, insulin tolerance test; HOMA-IR, homeostasis model assessment of basal insulin resistance; GLP-1, glucagon-like peptide 1. Data are presented as mean ± SD. ^#^*P* < 0.05, ^##^*P* < 0.01 vs. chow; ^*^P < 0.05, ^**^P < 0.01 vs. sham; ^&^*P* < 0.05, ^&&^*P* < 0.01 DJB vs. SG. *n* = 10 in each group.

Both sham and DJB groups exhibited higher body weight and food intake than the chow group, except when the rats were affected by surgical stress (Figures 1A,B). SG group had body weight and food intake similar to those of sham and DJB groups before surgery, but exhibited decreased food intake at 2 weeks post-operatively compared with the other three groups and lower body weight 3 weeks after operation than that in the sham and DJB groups (Figures 1A,B). No difference in body weight and food intake was observed between sham and DJB groups at any stage.

### Glucose Homeostasis After Surgery

One week after surgery, alleviation in FBG levels was observed in both DJB and SG groups as compared with the sham group, and the values reached to the level in the chow group at 4 weeks after operation (Figure 1C). OGTT and ITT was performed before surgery and 2 and 8 weeks after operation. The values of AUC_OGTT_ and AUC_ITT_ were lower in DJB and SG groups than in the sham group at 2 and 8 weeks post-operation (Figures 1D,E). The rats from DJB and SG groups exhibited lower HOMA-IR values at 2 and 8 weeks than those from the sham group (Figure 1F). These findings suggest that DJB and SG can reverse the impaired glucose tolerance and insulin sensitivity in diabetic rats thus improve glucose homeostasis. Moreover, the serum levels of GLP-1 in DJB and SG groups were higher than that of the sham group at 8 weeks after surgery (Figure 1G).

### Serum Lipid Profiles After Surgery

Lipid profiles were evaluated after operation to assess the effect of DJB and SG on lipid metabolism. As shown in Table 2, the rats from the sham group showed significantly impaired lipid profiles as compared with the chow group after 2 weeks of surgery. The levels of TG, TC, and NEFA in DJB and SG groups were lower than those in the sham group. These differences were also observed after 8 weeks of operation. The serum LDL level in DJB group decreased at 8 weeks after operation. No other difference was observed among the four groups.

**Table 2:** Serum lipid profiles after surgery.

	**2 weeks after surgery**				**8 weeks after surgery**			
	**Chow**	**Sham**	**DJB**	**SG**	**Chow**	**Sham**	**DJB**	**SG**
TG (mmol/L)	1.85 ± 0.33	2.33 ± 0.30^#^	1.87 ± 0.41^*^	1.85 ± 0.38^*^	1.87 ± 0.51	2.55 ± 0.46^#^	1.89 ± 0.42^*^	1.92 ± 0.44^*^
TC (mmol/L)	1.85 ± 0.44	2.51 ± 0.55^#^	1.82 ± 0.48^*^	1.89 ± 0.32^*^	1.82 ± 0.35	2.49 ± 0.55^#^	1.73 ± 0.41^**^	1.71 ± 0.48^**^
NEFA (μmol/dL)	33.60 ± 6.90	44.80 ± 7.13^#^	33.70 ± 9.66^*^	32.80 ± 9.51^*^	35.30 ± 7.59	46.10 ± 8.61^#^	32.40 ± 7.44^**^	31.60 ± 10.18^**^
HDL (mmol/L)	1.27 ± 0.32	1.13 ± 0.33	1.23 ± 0.22	1.17 ± 0.34	1.33 ± 0.30	1.22 ± 0.37	1.18 ± 0.24	1.15 ± 0.37
LDL (mmol/L)	0.27 ± 0.07	0.30 ± 0.07	0.31 ± 0.10	0.32 ± 0.10	0.28 ± 0.07	0.42 ± 0.11^#^	0.29 ± 0.11^*^	0.34 ± 0.13

*DJB, duodenal-jejunal bypass; SG, sleeve gastrectomy; TG, triglyceride; TC, total cholesterol; NEFA, non-esterified fatty acid; HDL, high-density lipoprotein; LDL, low-density lipoprotein. Data are presented as mean ± SD*.

# *P <0.05 vs. chow*;

* *P <0.05*,

** *P <0.01 vs. sham. n = 10 in each group*.

### Improvement in Lipid Metabolism in the Liver and WAT After DJB and SG

**Figure 2:**
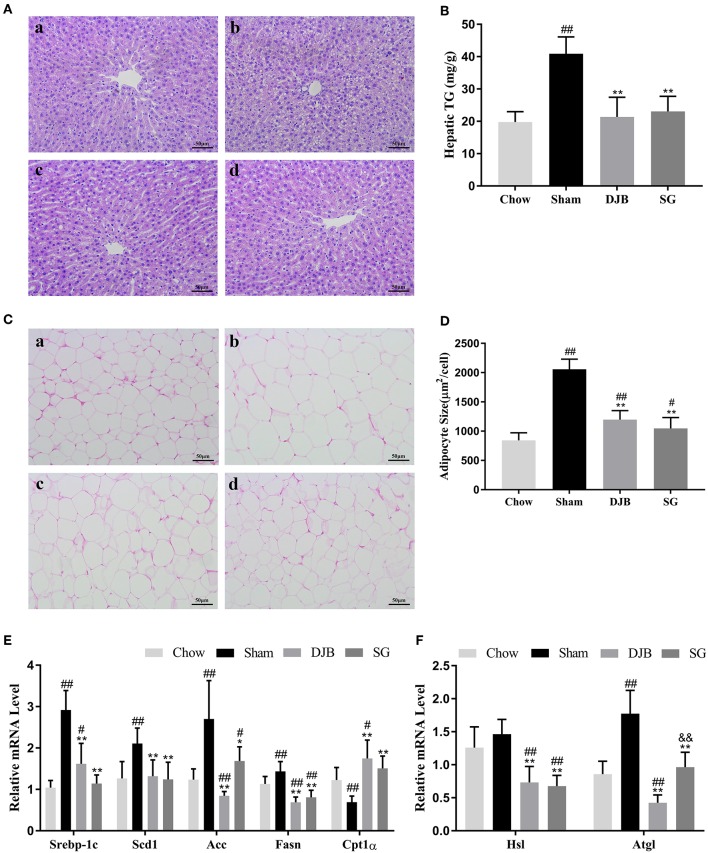
Improvement in lipid metabolism in the liver and WAT after DJB and SG. **(A)** HE staining of liver sections (scale bar, 50 μm); **(a)** chow; **(b)** sham; **(c)** DJB; **(d)** SG. **(B)** Hepatic TG level after surgery. **(C)** HE staining of the WAT section (scale bar, 50 μm); **(a)** chow; **(b)** sham; **(c)** DJB; **(d)** SG. **(D)** Adipose size after surgery. **(E)** Relative mRNA expression level of Srebp-1c, Scd1, Acc, Fasn, and Cpt1α in the liver. **(F)** Relative mRNA level of Hsl and Atgl in the WAT. DJB, duodenal-jejunal bypass; SG, sleeve gastrectomy; WAT, white adipose tissue; TG, triglyceride; Srebp-1c, sterol-regulatory element-binding protein 1c; Scd1, stearoyl-CoA desaturase 1; Acc, acetyl-CoA carboxylase; Fasn, fatty acid synthase; Cpt1α, carnitine palmitoyl-transferase 1α; Hsl, hormone-sensitive lipase; Atgl, adipose triglyceride lipase;. Data are presented as mean ± SD. ^#^*P* < 0.05, ^##^*P* < 0.01 vs. chow; ^*^P < 0.05, ^**^P < 0.01 vs. sham. *n* = 10 in each group. ^&&^*P* <0.01 DJB vs. SG.

DJB and SG groups exhibited significantly ameliorated lipid accumulation in the liver when compared with the sham group, as evident from HE staining (Figure 2A). In line with this result, the hepatic TG levels were downregulated in both DJB and SG groups as compared with the sham group (Figure 2B). Histological analysis of the adipose tissue revealed decreased adipocyte size in DJB and SG groups as compared with the sham group (Figures 2C,D). In the liver, the mRNA levels of sterol regulatory element-binding protein-1c (Srebp-1c), acetyl-CoA carboxylase (Acc), stearoyl-CoA desaturase 1 (Scd1), and fatty acid synthase (Fasn) decreased and the level of carnitine palmitoyl-transferase 1α (CPT1α) mRNA increased in DJB and SG groups as compared with those in the sham group (Figure 2E). We also observed downregulated expression of hormone-sensitive lipase (Hsl) and adipose triglyceride lipase (Atgl) in DJB and SG groups as compared with those in the sham group in the WAT (Figure 2F).

### Downregulation in the Serum Level of FGF21 and Hepatic FGF21 Synthesis After DJB and SG

**Figure 3:**
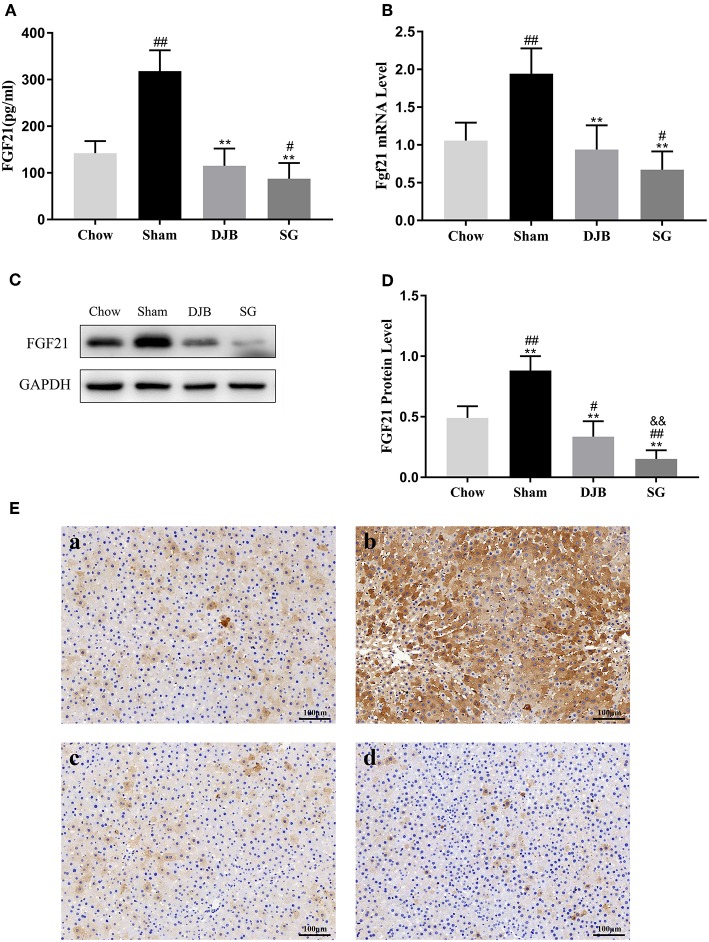
Decrease in the serum and hepatic levels of FGF21 after DJB and SG. **(A)** Serum FGF21 level (*n* = 10). **(B)** Relative mRNA expression of Fgf21 in the liver (*n* = 10). **(C,D)** Western blot analysis for hepatic FGF21 expression (*n* = 5). **(E)** Immunohistochemical images of FGF21 in liver sections (scale bar, 100 μm); **(a)** chow; **(b)** sham; **(c)** DJB; **(d)** SG. FGF21, fibroblast growth factor 21; DJB, duodenal-jejunal bypass; SG, sleeve gastrectomy. Data are presented as mean ± SD. ^#^*P* < 0.05, ^##^*P* < 0.01 vs. chow; ^**^P < 0.01 vs. sham; ^&&^*P* < 0.01 DJB vs. SG. *n* = 10 in each group.

As shown in Figure 3A, the level of circulating FGF21 increased in the sham group but significantly decreased after DJB and SG surgeries. As serum FGF21 is mainly derived through hepatic synthesis, we examined the expression of FGF21 in the liver. Consistent with the changes in the serum level of FGF21, both the mRNA and protein levels of FGF21 were elevated in the sham group but decreased in DJB and SG groups (Figures 3B–E). Interestingly, DJB and SG groups had lower FGF21 protein levels when compared with the chow group, and the protein level of FGF21 in SG group was significantly lower than that in DJB group (Figures 3C,D). Considering FGF21 is also a kind of myokine, we measured the weight of skeletal muscle (Table S1) and Fgf21 mRNA level in skeletal muscle (Figure S1) at 8 weeks after surgery and found no difference among the four groups.

### Restoration of the Impaired FGF21 Signaling Pathway in the Liver and WAT After DJB and SG

**Figure 4:**
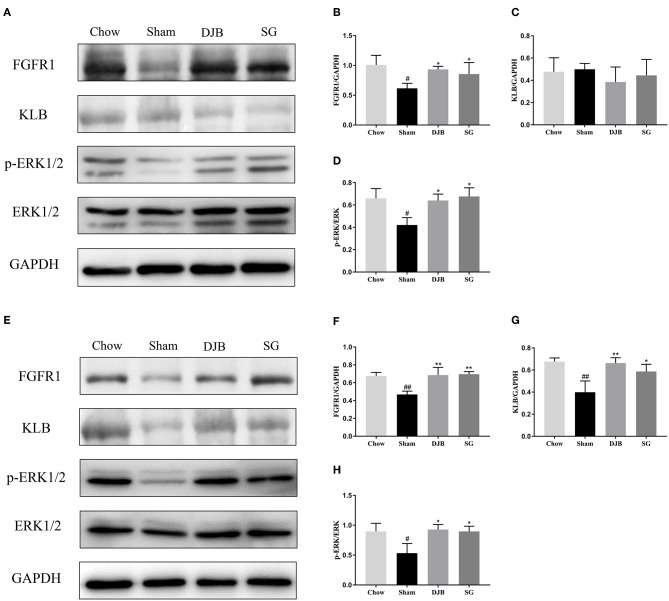
Restoration of FGF21 signaling pathway in the liver and WAT after DJB and SG. **(A–D)** Western blot analysis for the FGF21 signaling pathway in the liver. **(E–H)** Western blot analysis for the FGF21 signaling pathway in the WAT. FGF21, fibroblast growth factor 21; DJB, duodenal-jejunal bypass; SG, sleeve gastrectomy; FGFR, fibroblast growth factor receptor; KLB, β-klotho; ERK1/2, extracellular signal-regulated kinases 1 and 2; WAT, white adipose tissue. Data are presented as mean ± SD. ^#^*P* < 0.05, ^##^*P* < 0.01 vs. chow; ^*^P < 0.05, ^**^P < 0.01 vs. sham. *n* = 5 in each group.

The liver and WAT are two major targets of FGF21. We examined the changes in the FGF21 signaling pathway after DJB and SG surgeries. In both the liver and WAT, decreased expression of FGFR1 protein and reduced phosphorylation of ERK1/2 were observed in the sham group as compared with those in the chow group. After DJB and SG surgeries, the expression of FGFR1 and p-ERK1/2 increased to chow levels (Figures 4A,B,D–F,H). In addition, the KLB level decreased in the WAT from the sham group and was restored after DJB and SG surgeries (Figures 4E,G). However, no significant difference was observed in KLB level in the liver from the four groups (Figures 4A,C).

### Improved Response to Acute FGF21 Administration After DJB and SG

**Figure 5:**
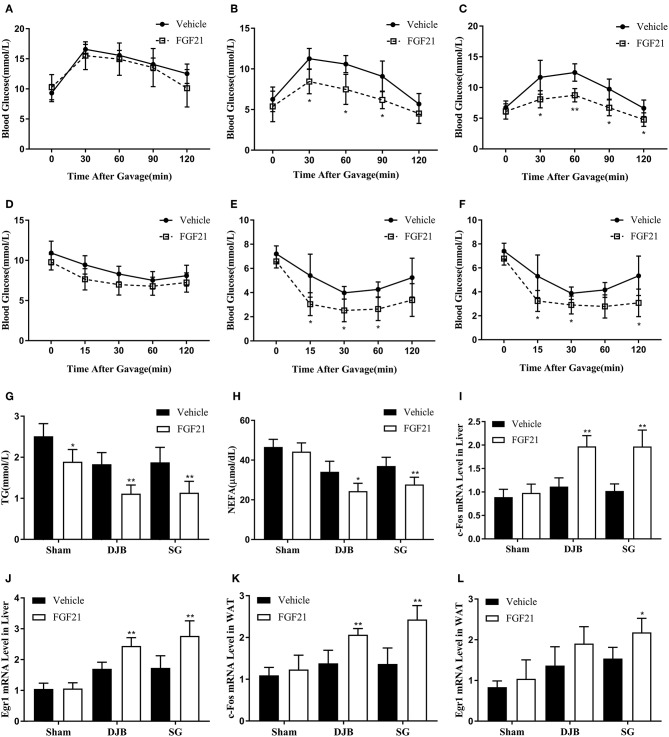
Improvement in response to acute FGF21 administration after DJB and SG. Recombinant human FGF21 or vehicle was administrated by a single intraperitoneal injection. OGTT and ITT were performed immediately after FGF21 injection. Blood samples, liver and WAT were harvested 2 h after FGF21 administration. **(A)** OGTT of sham group. **(B)** OGTT of DJB group. **(C)** OGTT of SG group. **(D)** ITT of sham group. **(E)** ITT of DJB group. **(F)** ITT of SG group. **(G)** Serum TG. **(H)** Serum NEFA. **(I)** c-Fos mRNA level in the liver. **(J)** Egr1 mRNA level in the liver. **(K)** c-Fos mRNA level in the WAT. **(L)** Egr1 mRNA level in the WAT. FGF21, fibroblast growth factor 21; DJB, duodenal-jejunal bypass; SG, sleeve gastrectomy; WAT, white adipose tissue; OGTT, oral glucose tolerance test; ITT, insulin tolerance test; TG, triglyceride; NEFA, non-esterified fatty acid; Egr1, early growth response gene 1. Data are presented as mean ± SD. ^*^P < 0.05, ^**^P < 0.01 vehicle vs. FGF21. *n* = 5 in each group.

To confirm whether bariatric surgery can improve FGF21 sensitivity, we then injected rhFGF21 to rats at 8 weeks post-operation to detect the response of the rats to FGF21. DJB and SG groups exhibited altered curves of OGTT and ITT after FGF21 administration (Figures 5B,C,E,F), whereas no significant alteration was observed in the sham group (Figures 5A,D). Downregulated serum TG and NEFA were also observed after FGF21 injection in DJB and SG groups (Figures 5G,H). We then examined the expression of early growth response 1 (Egr1) and c-Fos, the FGF21 target genes, after FGF21 injection. The mRNA levels of Egr1 and c-Fos were both shown to be upregulated in the liver and WAT of rats in DJB and SG groups after injection, with no significant changes observed in sham group (Figures 5I–L). These results demonstrate improved response to FGF21 administration after DJB and SG surgeries.

## Discussion

Bariatric surgery is a widely recognized approach to treat morbid obesity and T2DM (3, 4). In the present study, we reported decreased levels of circulating FGF21 along with significant downregulation of FGF21 expression in the liver after DJB and SG procedures. We also observed restored FGF21 signaling pathway and improved response to exogenous FGF21 administration in HFD/STZ-induced diabetic rats, suggesting improved FGF21 sensitivity after DJB and SG surgeries.

FGF21 is a newly recognized hormone with beneficial effects in both lipid and glucose metabolism (36). The paradoxical increase in FGF21 levels in obesity and diabetes has been reported in many studies (10, 19, 20), leading to the hypothesis of a potential FGF21-resistant state (21). In the present study, HFD in combination with low-dose STZ was used to induce diabetes in rats. This method is widely accepted to replicate the natural history and metabolic characteristics of patients with T2DM (37, 38). Many studies have confirmed the increase in the serum level of FGF21 in rats induced by HFD and STZ (39, 40). In line with these studies, we detected elevated serum FGF21 level in HFD/STZ-induced diabetic rats.

The effect of bariatric surgery on FGF21 level has been studied in several research groups. The level of circulating FGF21 after SG surgery is reported to be downregulated (31), whereas changes of serum FGF21 in DJB rats has not been reported so far. In the present study, we observed a significant decrease in the concentration of circulating FGF21 after both DJB and SG surgeries. DJB was originally designed to investigate the metabolic effect of intestinal rearrangement of RYGB. However, the effects of RYGB on FGF21 expression are discrepant. While some studies claim that FGF21 level is unaffected after RYGB (29, 30), others (25, 26) support the increase in FGF21 level after RYGB. Decreased level of circulating FGF21 was also observed after RYGB (27, 28). These conflicting results with RYGB is probably associated with its complicated weight-loss mechanism, including restricted gastric volume, altered intestine structure, and significant changes in gut hormone levels. Interestingly, the decrease in FGF21 level was also reported in other two forms of bariatric surgeries, namely, duodenal-jejunal omega switch (DJOS) that bypasses the entire duodenum and proximal jejunum (41), and ileal transposition (IT), a classic bariatric surgery with the transposition of a distal ileum segment to the proximal jejunum (42). All the four surgical techniques mentioned above (DJB, RYGB, DJOS, and IT) include the rearrangement of the small intestine. This similarity may suggest a potential role of small intestine in the regulation of FGF21 level after bariatric surgery.

Hepatic expression is the main part that contributes to the serum FGF21 level (10). In our study, we detected decreased expression of FGF21 in DJB and SG groups. And SG group exhibited lower protein level of FGF21 than DJB group. Considering FGF21 level is positively correlated with body mass index (43), and unlike SG, DJB is a bariatric surgical technique without weight reduction (44, 45), this difference in FGF21 expression between DJB and SG groups is probably attributed to the weight loss induced by SG. But since we also detected the decreased expression of FGF21 after DJB, the FGF21-lowering effect may partially, but not entirely, depend on weight loss.

FGF21 activates FGFR in the presence of KLB, phosphorylates ERK1/2, and regulates the downstream transcription to exert its function (17). KLB is a critical co-receptor that determines the action of FGF21 (18). Downregulated expression of KLB in the WAT has been reported in the FGF21-resistant state (22). In the present study, we observed decreased expression of KLB in the WAT of diabetic rats, which was restored after DJB and SG. However, we failed to detect any difference in KLB expression in the liver among the four groups. FGF21 binds to FGFR1-3, wherein FGFR1 seems to be the dominant receptor (46, 47). Consistent with the results of previous studies, we observed decreased level of FGFR1 in the liver and WAT of diabetic rats (19, 23, 24). FGFR1 level increased after DJB and SG as compared with that in the sham group. The impaired ERK1/2 phosphorylation in the adipose tissue in response to FGF21 has been reported in diet-induced obese mice (19, 21). In our study, the activation and phosphorylation of ERK1/2 were reduced in the sham group but the levels of p-ERK1/2 were elevated after DJB and SG. All these results are reflective of the enhancement in FGF21 signaling after DJB and SG surgeries. To our knowledge, this is the first study to demonstrate enhancement in FGF21 signaling after DJB and SG.

Since restored FGF21 signaling pathway was detected after DJB and SG surgeries, we then further confirmed the response of rats to the administration of exogenous FGF21 after surgery. In our study, we observed significant alterations after FGF21 administration in DJB and SG groups, but not in sham group. However, there is a general acknowledgment that FGF21 administration can improve glucose and lipid metabolism in obese and diabetic rodent (11). This discrepancy is probably because we only performed single injection of low-dose FGF21, while most studies with positive results were long-term FGF21 injection with relatively higher dose (11, 48). Actually in our study, we did observe significant decrease of serum TG in sham group after FGF21 administration. However, the alteration of other parameters in sham group was not significant enough. This low response to exogenous FGF21 is probably due to FGF21 resistance in obese and diabetic state, that low-dose FGF21 fails to exert significant corresponding effects. This phenomenon has been observed in previous study (21), which is also performed with single low-dose FGF21 administration. And the fact that DJB and SG groups can respond to low-dose FGF21 administration represents the improved FGF21 sensitivity after bariatric surgeries. DJB and SG groups exhibited altered glucose and lipid metabolism after FGF21 injection. Egr1 and c-Fos are the direct downstream molecules of ERK1/2 pathway (49), and are often used to monitor the FGF21 activity (50). The enhanced mRNA expression of Egr1 and c-Fos in DJB and SG groups reflects the activation of ERK1/2 pathway by FGF21 administration.

The detailed mechanism as to what leads to the restored FGF21 sensitivity after bariatric surgeries is unknown yet. A previous article reported that GLP-1 can induce the expression of FGFR and KLB in the liver and WAT thus improve FGF21 sensitivity (51). And administration of GLP-1 analog to T2DM patients and diet-induced obese mice can downregulate serum and hepatic FGF21 levels and decrease FGF21 resistance (52). In our study, we detected increased GLP-1 level 8 weeks after surgery in both DJB and SG groups. Taken together, GLP-1 probably played an important role in the improved FGF21 sensitivity after DJB and SG surgeries.

It remains unclear whether the restored FGF21 sensitivity contributes to the metabolic effects of DJB and SG surgeries. Morrison et al. (53) reported that RYGB is effective in FGF21 knockout mice, indicating that FGF21 is not a critical single factor for the beneficial effects of RYGB. But considering the great role of FGF21 in lipid and glucose metabolism, and the restored FGF21 sensitivity after DJB and SG reported in this study, we still can't rule out the possibility that the restored FGF21 sensitivity contributes to the improvements of bariatric surgery. Plasma FGF21 level positively correlates with serum and hepatic TG levels (11). In the present study, we observed decreased levels of TG, NEFA, and CHO, along with decreased lipid accumulation in the liver and WAT of both DJB and SG groups. FGF21 administration suppresses hepatic TG synthesis through inhibition of the transcription factor Srebp-1c and the downstream molecules including Scd1, Acc, and Fasn (48). In the present study, we observed downregulated expression of Srebp-1c, Scd1, Acc, and Fasn after DJB and SG surgeries, indicating the potential role of FGF21 in mediating the decrease in TG level. It was also reported that FGF21 can accelerate FFA oxidation in the liver (14) and inhibit lipolysis in the WAT of ob/ob mice, leading to a decrease in the plasma level of FFA (15). Consistent with these observations, we noted an upregulation in the expression of CPT1α in the liver and a decrease in the expression of Hsl and Atgl in the WAT of rats from DJB and SG groups, which may explain the decrease in the serum FFA level after surgery.

We acknowledge the limitations of the present study. Firstly, we didn't explore the effect of the improved renal clearance to the decreased serum FGF21 level after bariatric surgeries. FGF21 is mainly excreted by kidney (54), and serum FGF21 concentration is associated with the residual renal function in diabetic nephropathy (55). In present study (Table S2) and our previous studies (56, 57), renal function was impaired in sham group and restored after bariatric surgeries. So it's possible that the decreased serum FGF21 is partially due to the improved renal function. However, since liver is the main source of serum FGF21, and according to the downregulated FGF21 expression in the liver we observed after surgery, it is suffice to say that the downregulated hepatic FGF21 expression contributes to the decrease in serum FGF21. Secondly, though FGF21 may have connections with the metabolic improvements after bariatric surgeries, it is still insufficient to attribute the post-operative metabolic improvement to the restored FGF21 sensitivity, given that these metabolic improvements are also regulated by a lot of factors. Nevertheless, it is still meaningful to know that the FGF21 sensitivity is improved after bariatric surgeries, which gives us better understanding of the changes after bariatric surgeries and may provide possible strategies for the diabetic treatment.

In conclusion, DJB and SG surgeries can downregulate serum level and hepatic expression of FGF21, and restore FGF21 signaling pathway and FGF21 sensitivity in HFD/STZ-induced diabetic rats. The restoration in FGF21 sensitivity may be related to the post-operative metabolic improvements.

## Data Availability

All datasets generated for this study are included in the manuscript and/or the Supplementary Files.

## Ethics Statement

This study was carried out in accordance with the National Institutes of Health Guidelines. The protocol was approved by the Ethics Committee on Experimental Animals of Qilu Hospital of Shandong University.

## Author Contributions

SH and QL designed the experiments. QL, MW, XH, and YC performed the surgeries. QL, YS, PX, and SW performed the rest experiments and analyzed data. QL drafted the manuscript. MZ, SH, SL, and GZ edited and revised the manuscript.

### Conflict of Interest Statement

The authors declare that the research was conducted in the absence of any commercial or financial relationships that could be construed as a potential conflict of interest.
