# Supplementary material for: Improved FGF21 Sensitivity and Restored FGF21 Signaling Pathway in High-Fat Diet/Streptozotocin-Induced Diabetic Rats After Duodenal-Jejunal Bypass and Sleeve Gastrectomy
Source: Front Endocrinol (Lausanne). 2019 Aug 30;10:566. doi: 10.3389/fendo.2019.00566 (PMC6728857; doi:10.3389/fendo.2019.00566)
Supplement: Supplementary file 1 [file Data_Sheet_1.docx]

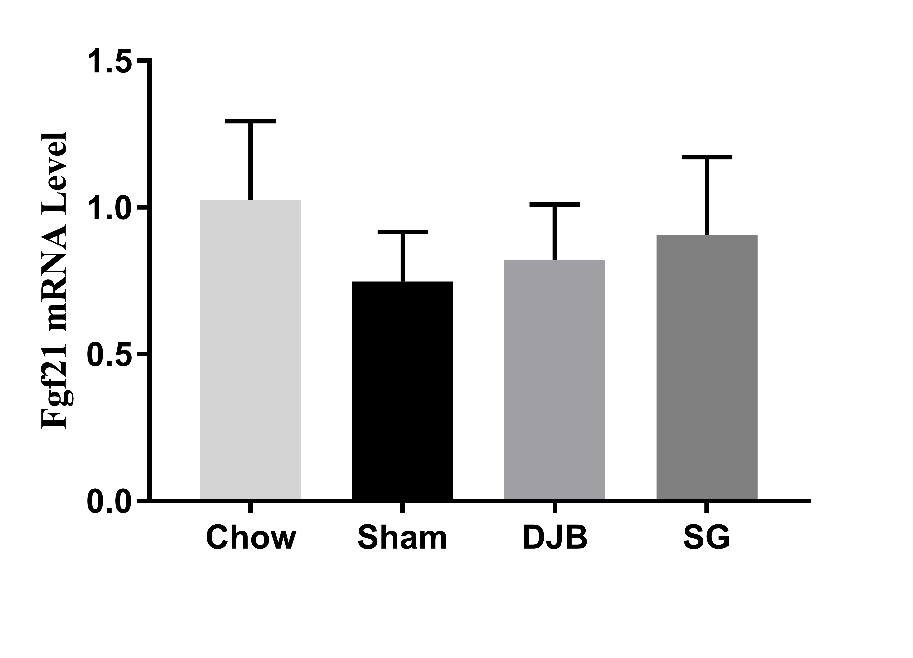


**Figure S1 Fgf21 mRNA level in skeletal muscle after surgery**

FGF21, fibroblast growth factor 21; DJB, duodenal-jejunal bypass; SG, sleeve gastrectomy. Data are presented as mean ± SD. n=10 in each group.

**Table S1 Relative skeletal muscle weight after surgery**

|  | Chow | Sham | DJB | SG |
| --- | --- | --- | --- | --- |
| muscle weight% | 0.46±0.10 | 0.50±0.08 | 0.53±0.08 | 0.47±0.06 |

DJB, duodenal-jejunal bypass; SG, sleeve gastrectomy. Data are presented as mean ± SD. n=10 in each group.

**Table S2 Serum creatinine and BUN after surgery**

|  | Chow | Sham | DJB | SG |
| --- | --- | --- | --- | --- |
| Creatinine | 2.69±0.76 | 5.70±0.57^##^ | 3.39±0.82^**^ | 2.44±0.94^**^ |
| BUN | 33.72±6.87 | 308.35±33.33^##^ | 35.74±7.61^**^ | 32.93±6.07^**^ |

BUN, blood urine nitrogen; DJB, duodenal-jejunal bypass; SG, sleeve gastrectomy. Data are presented as mean ± SD. n=10 in each group. ^##^P < 0.01 vs. chow; ^**^P < 0.01 vs. sham.
